# Infiltrating ductal carcinoma of the breast with coexisting lymphocytic mastitis in a non-diabetic adult female

**DOI:** 10.1259/bjrcr.20150234

**Published:** 2016-05-15

**Authors:** Jobin Mathew Jose, Anusha Varghese, George Joseph, Susmitha Keerthi, Jophy Varghese

**Affiliations:** ^1^ Department of Radiodiagnosis, Lourdes Hospital, Pachalam, Kochi, India; ^2^ Department of Pathology, Lourdes Hospital, Pachalam, Kochi, India

## Abstract

Lymphocytic mastitis, also known as diabetic mastopathy or sclerosing lymphocytic lobulitis, is a benign clinicopathological entity that, in earlier studies, has been described as an uncommon cause of breast mass in adult females with long-standing insulin-dependent diabetes mellitus. Further studies have suggested an autoimmune aetiology owing to its association with other autoimmune diseases such as Hashimoto's thyroiditis. On clinical examination, mammography and ultrasound, this lesion may mimic breast carcinoma. The most common mammographic findings are ill-defined masses or asymmetric densities. Such lesions are often masked by dense glandular tissue, making mammographic evaluation difficult. Ultrasound often reveals the characteristic finding of an irregular, hypoechoic mass with marked posterior acoustic shadowing. We present a case of infiltrating ductal carcinoma with coexisting lymphocytic mastitis involving the right breast of a non-diabetic adult female who presented with complaints of a painless, hard palpable lump in her right breast for 2 months. Mammography and ultrasonography showed features of a malignant lesion that was subjected to fine needle aspiration cytology and tru-cut biopsy examination. Cytology revealed features suggestive of infiltrating ductal carcinoma in a background of severe inflammation and necrosis. Tru-cut biopsy showed features suggestive of lymphocytic mastitis. The patient underwent modified radical mastectomy of the right breast. Histopathological examination of right breast tissue revealed multifocal infiltrating ductal carcinoma, metastatic ipsilateral axillary lymph nodes, lymphovascular tumour emboli and tumour-free margins. The patient underwent adjuvant chemotherapy and radiotherapy. She is on hormone therapy with a selective oestrogen receptor modulator and is disease-free now.

## Summary

Lymphocytic mastitis, also known as diabetic mastopathy or sclerosing lymphocytic lobulitis, is a benign clinicopathological entity, which in earlier studies has been described as an uncommon cause of breast mass in adult females with long-standing insulin-dependent diabetes mellitus.^1^ Further studies have suggested an autoimmune aetiology, owing to its association with other autoimmune diseases such as Hashimoto’s thyroiditis. On clinical examination, mammography and ultrasound, this lesion may mimic breast carcinoma.^2^ The most common mammographic findings are ill-defined masses or asymmetric densities. Such lesions are often masked by dense glandular tissue, making mammographic evaluation difficult. Ultrasound often reveals the characteristic finding of an irregular, hypoechoic mass with marked posterior acoustic shadowing.^3^

We present a case of infiltrating ductal carcinoma with coexisting lymphocytic mastitis involving the right breast of a non-diabetic adult female who presented with complaints of a painless, hard palpable lump in her right breast for 2 months. Mammography and ultrasonography showed features of a malignant lesion that was subjected to fine needle aspiration cytology and tru-cut biopsy examination. Cytology revealed features suggestive of infiltrating ductal carcinoma in a background of severe inflammation and necrosis.

Tru-cut biopsy showed features suggestive of lymphocytic mastitis. The patient underwent modified radical mastectomy of the right breast. Histopathological examination of right breast tissue revealed multifocal infiltrating ductal carcinoma, metastatic ipsilateral axillary lymph nodes, lymphovascular tumour emboli and tumour-free margins. The patient underwent concurrent chemoradiotherapy. She is on oral hormone therapy with tamoxifen (a selective oestrogen receptor modulator) and is disease-free now.

## Clinical presentation

A 47-year-old female patient with no comorbid disease presented to our hospital with a painless, hard palpable lump in her right breast that she first noticed 2 months ago.

## Investigations/imaging findings

**Figure 1.**
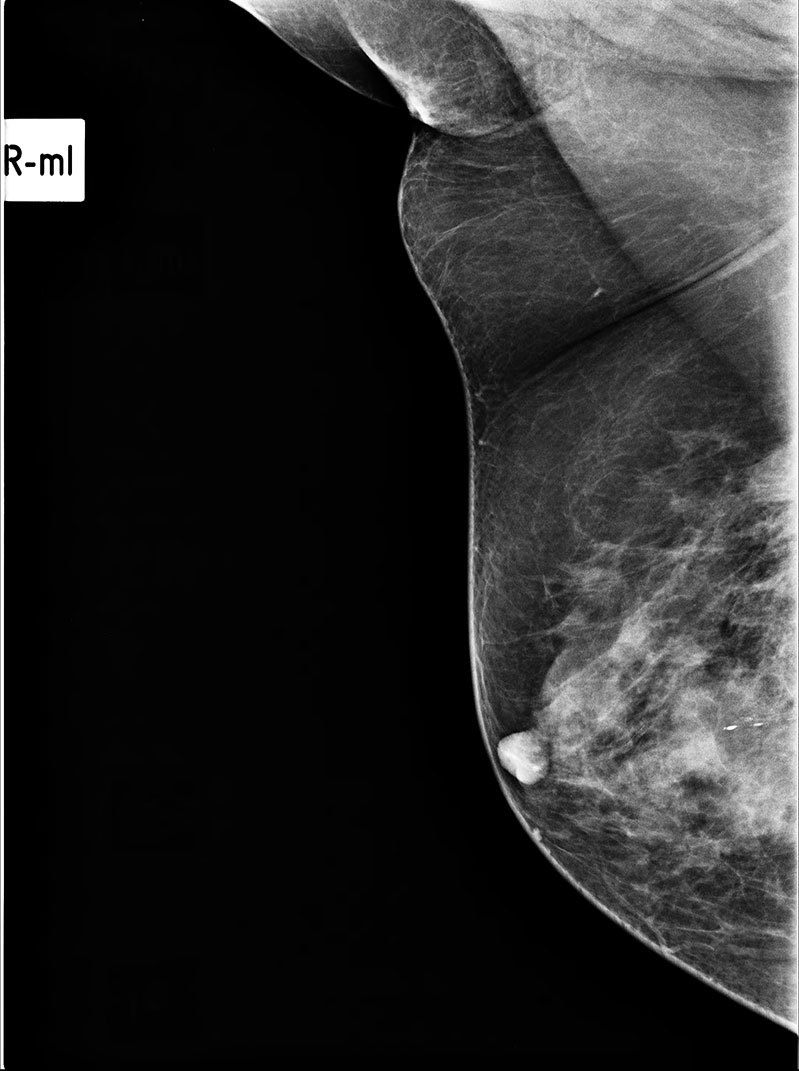
Right breast mammogram, mediolateral oblique view. A focal asymmetrical density is seen in the right subareolar region.

**Figure 2.**
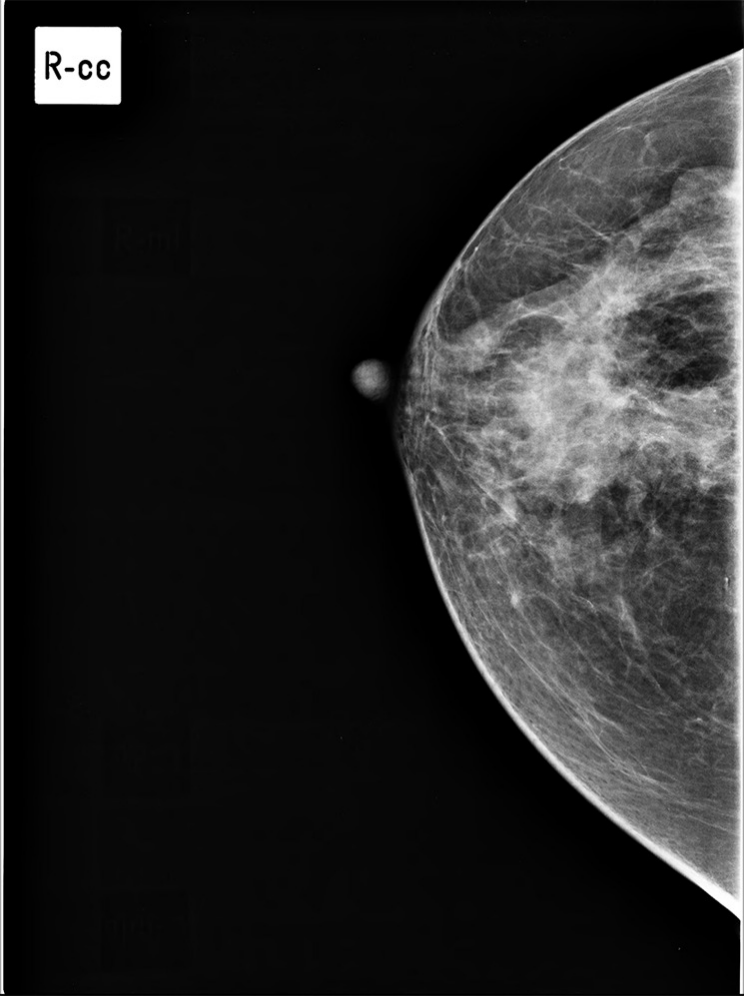
Right breast mammogram, craniocaudal view. A focal asymmetrical density is seen in the right subareolar region.

**Figure 3.**
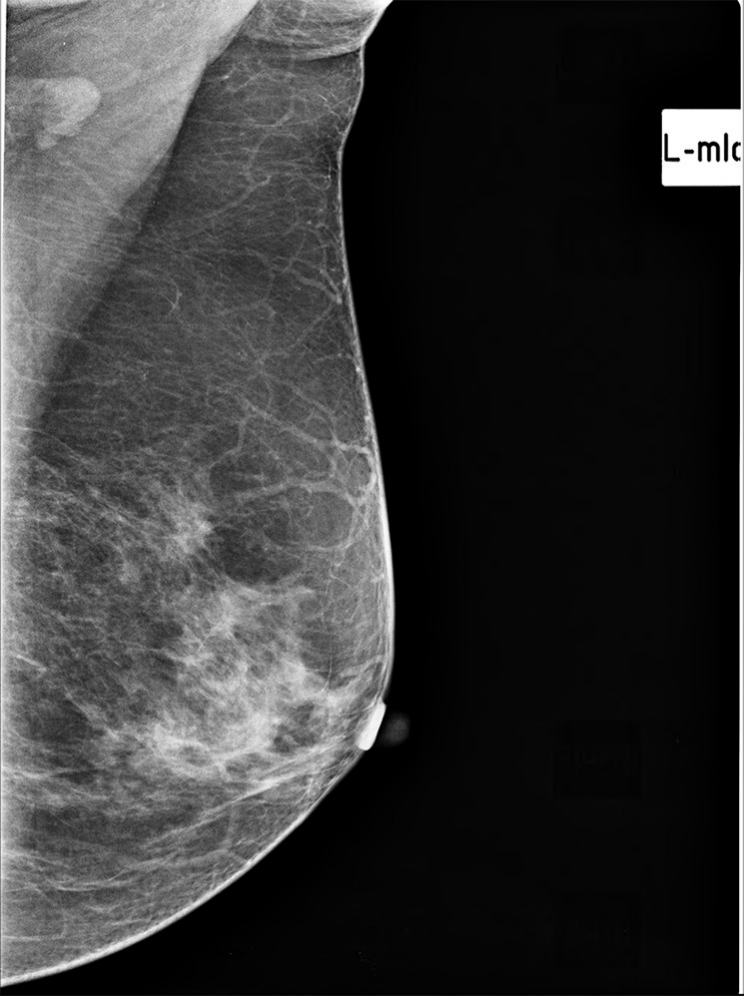
Left breast mammogram, mediolateral view. No evidence of focal asymmetrical density or mass lesion within. A mildly enlarged benign-appearing lymph node in the left axilla.

**Figure 4.**
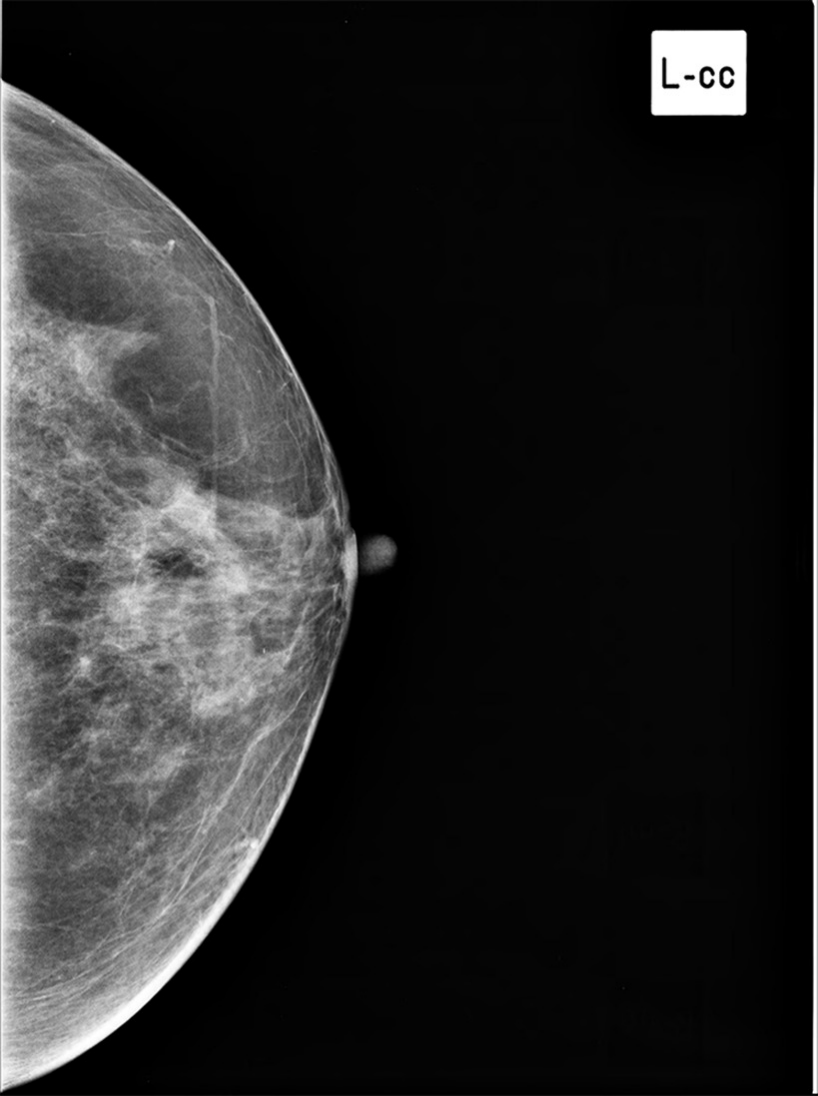
Left breast mammogram, craniocaudal view. No evidence of focal asymmetrical density or mass lesion within.

Mammography showed heterogeneous glandular parenchyma with focal asymmetric density in the subareolar region of the right breast. Another focal asymmetric density with microcalcific foci was present in the right retromammary area (Figures 1–4).

**Figure 5.**
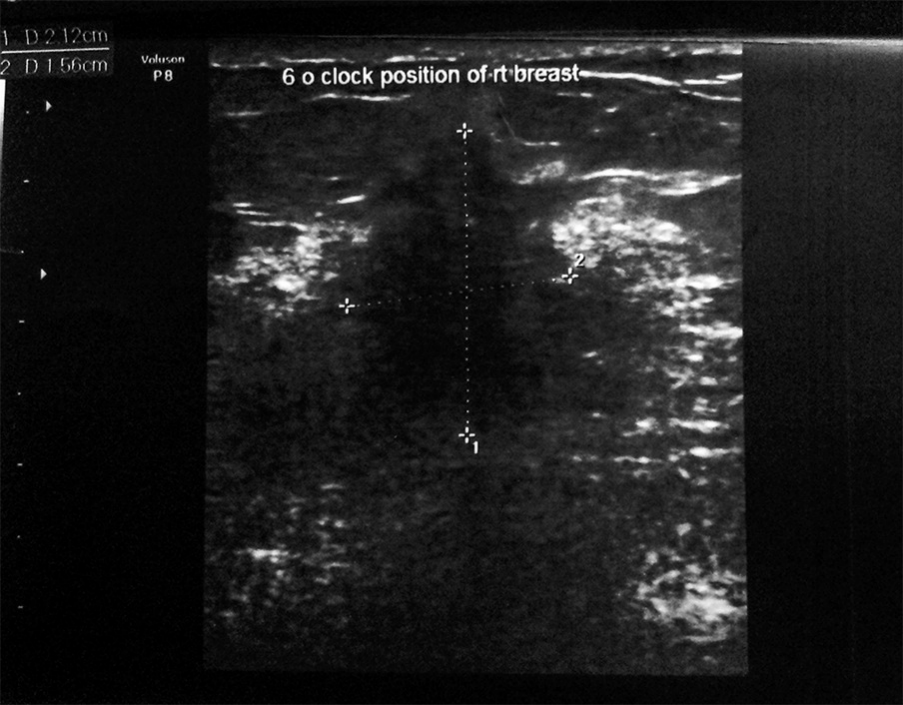
Right breast ultrasound. An ill-defined, hypoechoic, taller-than-wide lesion measuring 2.1 × 1.5 cm at the 6 o'clock position in the right breast.

Ultrasonography of the right breast showed an ill-defined, hypoechoic, taller-than-broad lesion measuring 2.5 × 1.5 cm with few microcalcific foci within (Figure 5). Few other ill-defined hypoechoic lesions were also seen in the inferomedial quadrant of the right breast. Mammography and sonography were suggestive of a breast imaging-reporting and data system category 4C lesion.

Ultrasound-guided fine needle aspiration cytology of the lesion showed highly cellular smears with clusters of malignant cells having hyperchromatic nuclei and highly irregular coarse chromatin in a background of necrosis and severe inflammation. Mitotic features were also seen. The cytological features were suggestive of an infiltrating ductal carcinoma.

Ultrasound-guided tru-cut biopsy from the same lesion showed features of lymphocytic mastitis with dense periductal, intralobular and perilobular infiltrates of small lymphocytes. Mild ductal epithelial hyperplasia was also seen, with no evidence of malignancy.

Ultrasonography of the abdomen showed diffuse mild fatty infiltration of the liver, with no focal lesions within.

Histopathological examination of the mastectomy specimen showed a diffusely infiltrating irregular tumour, with tumour cells showing eosinophilic cytoplasm, pleomorphic and hyperchromatic nuclei with prominent nucleoli. Foci of necrosis and intermediate-to-high grade ductal carcinoma *in situ* were also seen. The surrounding stroma showed inflammatory infiltrates. The deep resection margins were free of tumour. Metastatic ipsilateral axillary lymph nodes and lymphovascular tumour emboli were present. Based on these findings, the tumour was staged as pT2N3aMx.

## Differential diagnosis

Histopathologically, a prominent lymphoplasmacytic background can be seen in breast carcinomas. Studies have also suggested that a probable immune response against breast tumours can cause lymphocyte proliferation, which is seen in early-onset breast carcinoma. This has a prognostic significance, as these patients can be selected for active immunotherapy because they are immunologically responsive.^4^

Lymphocytes in benign lymphocytic mastitis and breast carcinomas appear similar morphologically, with prominent lymphocytic infiltrates in the breast ducts and lobules, and the perivascular regions. Core biopsies can sometimes reveal only prominent lymphocytic proliferation, particularly if the site immediately adjacent to the tumour is sampled.

Differential diagnosis of lymphocytic mastitis includes breast carcinoma, granulomatous lobular mastitis and fibrotic tissue.^5^ Granulomatous lobular mastitis is commonly unilateral and most often seen in the periphery of the breast. Its most common mammographic appearance is a focal asymmetric density with indistinct margins. The most frequent sonographic finding is an irregular hypoechoic mass with multiple tubular hypoechoic finger-like extensions.^6^

## Treatment

The patient underwent modified radical mastectomy of the right breast. On the basis of the histopathology report, adjuvant concurrent systemic chemotherapy and regional radiotherapy were started. The patient received six cycles of chemotherapy (CMF regimen—cyclophosphamide, methotrexate and 5-fluorouracil), which she tolerated well. Oral hormone therapy was started (after chemotherapy) with tamoxifen, a selective oestrogen receptor modulator.

## Outcome and follow-up

The patient responded well to concurrent chemoradiotherapy. She had no complaints at her regular follow-up visits. No abnormality was detected on physical examination or breast ultrasound, except for benign postoperative changes.

## Learning points

1. Although lymphocytic mastitis is usually associated with long-standing poorly controlled insulin-dependent diabetes mellitus, it can occur in non-diabetic patients as well as in association with other autoimmune diseases.
2. Lymphocytic mastitis can mimic or coexist with breast carcinoma clinically and radiologically.^7^
3. Proliferating lymphocytes share the same morphology in benign lymphocytic mastitis, as well as in breast carcinomas.
4. Pathological diagnosis of lymphocytic mastitis on image-guided biopsy samples should alert the clinician to the possibility of underlying malignancy, particularly in patients who are clinically and radiologically suspected to have a malignant breast lesion.
